# Manganese-based type I collagen-targeting MRI probe for in vivo imaging of liver fibrosis

**DOI:** 10.21203/rs.3.rs-5349052/v1

**Published:** 2024-11-22

**Authors:** Chunxiang Zhang, Hua Ma, Daniel DeRoche, Eric M. Gale, Pamela Pantazopoulos, Nicholas J. Rotile, Himashinie Diyabalanage, Valerie Humblet, Peter Caravan, Iris Y. Zhou

**Affiliations:** 1 Athinoula A. Martinos Center for Biomedical Imaging, Institute for Innovation in Imaging (i^3^), Department of Radiology, Massachusetts General Hospital, Harvard Medical School, Boston, USA.; 2 Collagen Medical LLC, Boston, USA.

**Keywords:** collagen, manganese, fibrosis, molecular imaging, MRI

## Abstract

Liver fibrosis is a common pathway shared by all forms of progressive chronic liver disease. There is an unmet clinical need for noninvasive imaging tools to diagnose and stage fibrosis, which presently relies heavily on percutaneous liver biopsy. Here we explored the feasibility of using a novel type I collagen-targeted manganese (Mn)-based MRI probe, Mn-CBP20, for liver fibrosis imaging. In vitro characterization of Mn-CBP20 demonstrated its high binding affinity for human collagen (*K*_d_ = 9.6 μM), high T_1_-relaxivity (48.9 mM^−1^s^−1^ at 1.4T and 27°C), and kinetic inertness to Mn release under forcing conditions. We demonstrated MRI using Mn-CBP20 performs comparably to previously reported gadolinium-based type I collagen-targeted probe EP-3533 in a mouse model of carbon tetrachloride-induced liver fibrosis, and further demonstrate efficacy to detect fibrosis in a diet-induced mouse model of metabolically-associated steatohepatitis. Biodistribution studies using the Mn-CBP20 radio-labeled with the positron-emitting ^52^Mn isotope demonstrate efficient clearance of Mn-CBP20 primarily via renal excretion. Mn-CBP20 represents a promising candidate that merits further evaluation and development for molecular imaging of liver fibrosis.

## Introduction

Chronic liver disease (CLD) is a major global health burden that contributes to approximately 2 million deaths annually worldwide^1^. Sustained insult resulting from conditions including hepatitis B or C virus infection, fatty liver disease, alcohol use, toxins, and autoimmune and genetic disorders, can result in chronic activation and dysregulation of tissue repair mechanisms leading to liver fibrosis^2,3^. Regardless of the underlying cause, liver fibrosis is a common pathological feature of all progressive liver disease and associates directly with poor outcomes including cirrhosis, hepatocellular carcinoma, and liver failure^4,5^. On the other hand, the liver has the capacity to reverse fibrosis if the underlying cause is eliminated^6^. In this regard, liver fibrosis assessment is key to prognosticating CLD outcomes and to evaluating response to therapy. Liver biopsy is the current gold standard for diagnosing and staging liver fibrosis, but the procedure is invasive and carries non-negligible risks of pain, bleeding, and infection, and samples online a small portion of the liver^7^. Thus, there is an unmet need for noninvasive imaging to detect, tomographically map, quantify, and surveil the progression and resolution of liver fibrosis.

Serum biomarker panels and clinical imaging modalities such as ultrasound and magnetic resonance (MR) elastography are generally effective for detection of advanced fibrosis but perform less reliably in diagnosis and staging earlier fibrosis^8–10^. Molecular MR imaging using probes targeted to type I collagen, a protein that is abundantly present in the extracellular matrix of fibrotic tissue, has shown promise in experimental studies to detect and quantify liver fibrosis in animal models^11–14^. For example, MRI using the gadolinium (Gd)-based type I collagen-targeted probe EP-3533 was shown to provide higher sensitivity than MR elastography to diagnose early-stage liver fibrosis in a rat model of diethylnitrosomine-induced liver fibrosis^14^. Unfortunately, long-term retention of Gd following contrast-enhanced MRI examinations is well documented^15^, and concerns related to residual Gd represent substantial hurdles to the development and adoption of new Gd-based imaging probes.

In this study, we explored the feasibility of using a novel type I collagen-targeted manganese (Mn)-based MRI probe, Mn-CBP20, for liver fibrosis imaging. We demonstrate high affinity for type I collagen and high relaxivity similar to EP-3533, demonstrate how MRI using Mn-CBP20 performs comparably to EP-3533 in a mouse model of carbon tetrachloride (CCl_4_)-induced liver fibrosis, and further demonstrate efficacy to detect fibrosis in a choline-deficient, L-amino acid-defined, high-fat diet (CDAHFD) mouse model of metabolically-associated steatohepatitis (MASH). Biodistribution studies using the Mn-CBP20 radio-labeled with the positron-emitting ^52^Mn isotope demonstrate efficient clearance of Mn-CBP20 primarily via renal excretion.

## Materials and methods

### Synthesis of MnCBP20.

A batch of 0.204 g (0.091 mmol) collagen-binding peptide CM101P was stirred in 6 mL DMF. To this mixture, a few drops of DIPEA were added so that pH check on wetted pH paper returned pH ≥ 8.5 and the solution turned from heterogenous to clear and homogenous. Next, a total of 149 mg (0.45 mmol) CDTA-monoanhydride was added in three equal portions (48, 52, and 49 mg each) roughly 1 hour apart and the reaction was monitored by LC-MS. Addition of CDTA-monoanhydride initially resulted in a heterogenous white mixture, which becomes clear and homogenous upon conversion to the tri-acetylated intermediate. The DMF solution was added dropwise into 95 mL EtOAc and the resultant white solids were isolated by centrifugation and decanting the supernatant. The crude solids were next taken up in 20 mL water and then 90 mg MnCl_2_•4H_2_O (0.45 mmol) were added and the solution was adjusted pH 7 using NaOH. Any solids were removed by filtration and the mixture was purified by reverse phase HPLC using a Teledyne IPSCO RediSep Gold C18 column. Mobile phase A: water adjusted to pH 7.0, Eluent B: Acetenitrile. Gradient program: 5% B for 2 column volumes, ramp from 5 to 50% B over 8 column volumes, hold at 50% B for 4 column volumes. Fractions containing pure Mn-CBP20 were pooled and lyophilized to obtain 110 mg (0.033 mmol, 36% yield) of Mn-CBP20 as white solids. ESI-MS: m/2z = 1693.2 [M+5H]^2+^; calcd. 1693.1.

### Relaxivity measurements.

Relaxivity measurements were performed on a Magritek Spinsolve 60 MHz Multi-X Plus NMR spectrometer, 1.41T and 27 °C. Longitudinal (T_1_) relaxation was acquired via an inversion recovery measurement and transverse (T_2_) relaxation was measured using a Carl-Purcell-Meibooom-Gill spin echo measurement. Relaxivity (r_1,2_) was determined from the slope of the plot of 1/T_1,2_ vs. probe concentration for at least 4 concentrations.

### Collagen binding affinity assay.

Mn-CBP binding affinity to human type I collagen was evaluated by incubating varied concentrations of the probe with type I collagen deposited on a well plate or in a blank well for 2 hours as described previously^12,16^. The same experiment using EP-3533 was performed side by side for comparison. Mn and Gd concentration in the blank well and in the collagen-containing well were measured by ICP-MS to determine the total probe concentration and unbound probe concentration respectively. The difference in total and unbound probe concentration corresponds to the concentration of probe that is bound to collagen. To estimate binding affinity, the data were plotted and fit according to the following equation

$$\frac{\left[bound\right]}{\left[collagen\right]}=\frac{N[free]}{K_{d}+[free]}$$

Where N corresponds to the number of binding sites per collagen monomer and $K_{d}$ corresponds to the dissociation constant. Here N was fixed to 2.

### [^52^Mn]Mn-CBP20 radiolabeling.

An aliquot of 70.3 MBq ^52^MnCl_2_ in 0.3 mL HCl (0.1 M) (t_1/2_ = 5.6 days, obtained from the Cyclotron Facility at the University of Alabama, Birmingham, AL) was spiked to 1 mL stock solution of Mn-CBP20 (6 mM, pH 6–7). The pH was carefully adjusted to pH 3 by adding 1 M HCl and stirred at room temperature for 1 hour, and then the pH was adjusted back to pH 6 to 7 by adding 1 M NaOH. The volume of added HCl and NaOH was recorded, and the total volume was then brought to 2 mL accordingly by adding sterile saline.

### Animal Models.

All animal experiments were performed in accordance with the NIH Guide for the Care and Use of Laboratory Animals and in compliance with the ARRIVE guidelines ^17^ and approved by the MGH Institutional Animal Care and Use Committee. A total of 38 C57BL/6 mice (Charles River Laboratories, Wilmington, MA) were studied. All the mice were housed in an animal facility under controlled temperature and 12-hour light/12-hour dark cycle conditions.

#### CCl_4_-induced liver fibrosis:

Male C57BL/6 mice (6-week-old) were treated with an oral gavage of carbon tetrachloride for 6 weeks (N = 5) or 12 weeks (N = 5) (2–3 times per week, 0.1 mL of 20% CCl_4_ in olive oil the first week, 30% the second week, and 40% from weeks 3 through 6 or 12). Control mice were treated with vehicle (olive oil) only for 6 weeks (N = 5) or 12 weeks (N = 3). In the 12-week CCl_4_ group, there was 1 death due to accidental instillation of CCl_4_ into the trachea, and 1 death at the 10^th^ week, leaving 3 mice undergoing MRI and tissue analysis. Animals treated with CCl_4_ or vehicle for 6 weeks were imaged with Mn-CBP20 (10 μmol/kg). Animals treated with CCl_4_ or vehicle for 12 weeks were imaged with Mn-CBP20 (10 μmol/kg) and EP-3533 (10 μmol/kg) on 2 consecutive days in a random order.

#### CDAHFD model of NASH:

To induce MASH, 6-week-old, male C57BL/6 mice were fed CDAHFD (N = 5) consisting of 60 kcal% fat and 0.1% methionine by weight (A06071302; Research Diets, New Brunswick, NJ) for 6 weeks. Another group of gender and age-matched mice (N = 3) were fed standard chow for 6 weeks.

#### Biodistribution and elimination:

An additional group of 20 adult C57BL/6 mice (10–12 weeks old, 10 males and 10 females) received Mn-CBP20 radiolabeled with Mn-52. Each mouse was administered a cocktail dose of ^52/nat^Mn-CBP20 (^52^Mn complex: 2.3–3.1 MBq, ^nat^Mn complex: 10 μmol/kg body weight). After injection, mice were housed in cages with bedding changed every 8 h to prevent reabsorption of excreted dose. At day 1 and day 7 post-injection, respectively, 5 male and 5 female mice underwent PET-CT and were euthanized to evaluate biodistribution and elimination of the probe.

### MRI data acquisition and analysis.

Animals were anesthetized with isoflurane (3%) and placed in a specially designed cradle with body temperature maintained at 37°C. The isoflurane level was maintained at 1–2% for a respiration rate of 60 ± 5 breaths per minute. Imaging was performed using a 4.7 Tesla MRI scanner (Bruker, Billerica MA) with a custom-built volume coil. The tail vein was cannulated for i.v. delivery of the MRI probe. A series of three-dimensional (3D) T1-weighted fast low-angle shot (FLASH) MR images were first acquired (repetition time/echo time = 15/2 ms; field of view = 48 mm x 30 mm x 25 mm; matrix = 192 × 120 × 100; flip angle = 30°; acquisition time = 3 min), then a bolus of molecular probe was administered intravenously, and imaging was repeated with the 3D T1-weighted FLASH for 45 min. After the imaging session, animals were sacrificed (90 min after injection), and the liver tissue was subjected to histopathologic analysis.

The liver-to-muscle contrast-to-noise ratio (CNR) was calculated as the difference in signal-to-noise ratio between liver and muscle regions of interest (ROI). An ROI was manually traced, encompassing the liver parenchyma while avoiding major blood vessels. A second ROI was placed on the dorsal muscle visible in the same image slice to quantify the signal intensity in the muscle for comparison. The third ROI was placed in the field of view without any tissue (air) to measure the variation in the background signal. The same analysis was performed on the pre-and post-injection images acquired with the FLASH sequence. Image visualization and quantification were performed in AMIDE^18^. CNR was calculated by subtracting the signal intensity (SI) in the muscle from that in the liver and normalizing to the standard deviation (SD) of the signal in the air outside the animal, $\text{CNR}=\left({\text{SI}}_{\text{liver}}-{\text{SI}}_{\text{muscle}}\right)/{\text{SD}}_{\text{air}}$. ΔCNR was calculated by subtracting the ${\text{CNR}}_{\text{pre}}$ from ${\text{CNR}}_{\text{post}}$, $\text{ΔCNR}={\text{CNR}}_{\text{post}}-{\text{CNR}}_{\text{pre}}$. The dynamic MRI signal time courses of the liver were calculated by measuring the percentage change in signal intensity between post-injection and pre-injection images.

### PET-CT imaging.

Mice were imaged in a MultiScan^™^ LFER 150 PET/CT scanner (Mediso USA, LLC; Arlington, VA, USA). Static PET data were acquired in list-mode and were reconstructed using the 3D ordered subset expectation maximization (OSEM) image reconstruction algorithm with 30 iterations and 1 subset (Tera-Tomo 3D) and were corrected for scatter and attenuation. Image visualization and quantification were performed in Horos (Horosproject.org, Nimble Co LLC d/b/a Purview, Annapolis, MD, USA.)

### Tissue analysis.

After imaging, the animals were sacrificed under anesthesia, and the liver tissues were harvested. A piece of the left lobe of the liver was fixed in 4% paraformaldehyde in PBS, dehydrated, embedded in paraffin, and then sectioned into 5-μm-thick slices for later staining with Sirius Red and hematoxylin and eosin (H&E). Another piece of the left lobe was quickly frozen in liquid nitrogen for hydroxyproline analysis. The collagen proportional area (CPA), as determined by the percentage area stained with Sirius Red, was quantified from the histologic images by using ImageJ ^19^. Hydroxyproline in tissue was quantified with high-performance liquid chromatography analysis of tissue acid digests as previously described ^20^. Hydroxyproline is expressed as the amount per wet weight of tissue.

### Ex vivo Mn-52 quantification.

Ex vivo quantification of ^52^Mn activity in each injected dose was determined using a Capintec CRC-15PET dose calibrator. Injected dose activity was determined from the difference in activity recorded in the loaded syringe and injection line prior to and after injection. This value was then adjusted by subtracting the activity measured from the injection site (tail). Quantification of Mn-52 activity in ex vivo tissue samples was performed using a Perkin Elmer 2480 Wizard2 gamma counting system. Percentage injected (%ID) dose in ex vivo tissue samples was determined by comparing tissue counts to a measured aliquot of the injected dose. Bone, blood, fat, and muscle were estimated to account for 13%, 7%, 13%, and 40% of the total body weight. All measurements were corrected to account for decay.

### Statistical analysis.

Data are displayed as box plots with the dark band inside the box representing the mean, the bottom and top of the box representing the first and third quartiles, and the whiskers representing the minimum and maximum values. Data are reported as the means ± SD. Differences between the two groups were tested with a two-tailed paired or unpaired t-test. Differences among more than two groups were tested with one-way analysis of variance (ANOVA), followed by Tukey’s post hoc test with P < 0.05. Correlations between CPA and MR imaging metrics (ΔCNR) of fibrosis were assessed with the Pearson correlation coefficient. P values less than 0.05 were considered to indicate statistical significance. All statistical analyses were performed using GraphPad Prism 10 (GraphPad software).

## Results

### Mn-CBP20 Design and Synthesis.

We sought a Mn-based imaging probe construct that binds type I collagen specifically and with high avidity, is thermodynamically stable and kinetically inert with respect to Mn release, and can be manufactured in as few synthetic steps as possible. For targeting of type I collagen we utilized the peptide CM101P, which is based on collagen-specific cyclic decapeptide sequences identified through a phage display screen and has been further optimized for collagen binding^16^. CM101P is the same disulfide bridged cyclic decapeptide with flanking amino acids that was employed in the previously reported collagen-targeted probes EP-3533, CM-101, and SNIO-CBP^16,21,22^. To tightly incorporate Mn^2+^ as the MR signal-generating component, the peptide was conjugated with three CDTA-monoamide ligands. Prior studies have demonstrated how hexadentate ligands built from the rigid trans-1,2-diaminocylcohexane core bind Mn^2+^ with high-thermodynamic stability and are kinetically inert to Mn^2+^ de-chelation.^23–28^ Furthermore, the CDTA-monoanhydride synthon used for ligand conjugation can be generated cleanly and quantitatively from inexpensive materials in a single step.

**Figure 1.**
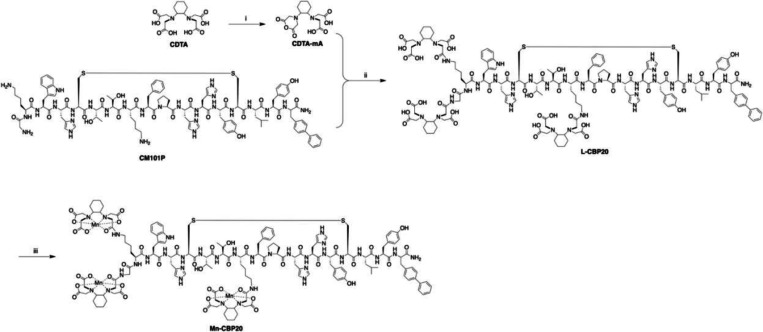
Synthesis of Mn-CBP20. (i) acetic acid, pyridine, RT, 38 h; (ii) CDTA-mA, DMF, DIPA, RT; (iii) MnCl_2_•4H_2_O, pH 7 H_2_O.

The synthesis of Mn-CBP20 is outlined in Figure 1. Briefly, CDTA-monoanhydride was synthesized from commercially available CDTA by stirring in acetic anhydride with pyridine added, as reported previously,^29^ and then conjugated with CBP via stirring in DMF with DIPEA. Next, the crude reaction product containing L-CBP20 was reacted with MnCl_2_ and Mn-CBP20 isolated after purification RP-HPLC in 36% overall yield based on CM101P.

### Mn-CBP20 physical properties are well suited for molecular MR imaging of collagen.

**Figure 2.**
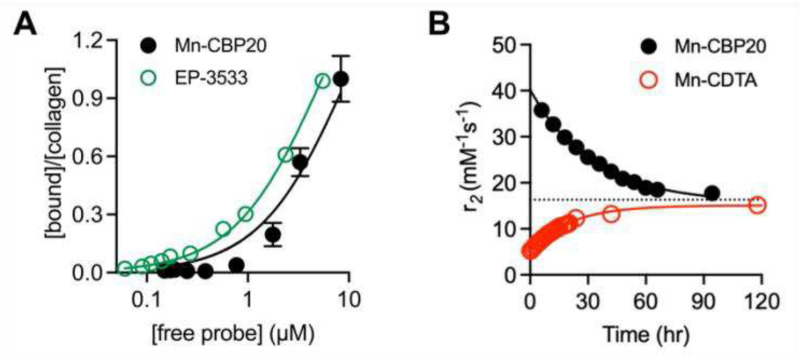
In vitro assays for collagen binding and kinetic inertness. **(A)** Plots of free vs collagen bound Mn-CBP20 (black circles) and EP-3533 (open green circles) demonstrate comparable affinity for human type I collagen at RT. **(B)** Kinetics of Mn^2+^ dissociation from Mn-CBP20 (black circles and Mn-CDTA (open black circles) under forcing conditions (0.5 mM Mn2+, 10 mM Zn(OT)_2_, pH 7.4 50 mM Tris: 18 mM citrate, 27 °C) monitored by r_2_ change at 1.4T and indicates Mn-CBP20 is more kinetically inert than Mn-CDTA. The dotted black line corresponds to the r_2_ value of MnCl_2_•4H_2_O dissolved in the corresponding buffer mixture.

Mn-CBP20 binds to human collagen with comparable affinity to the previously reported probe EP-3533, Figure 2A. The collagen affinity of Mn-CBP20 was similar to that of EP-3533 with ${\text{K}}_{\text{d}}$ values of 9.5 ± 2.5 μM and 5.5 ± 1.1 μM, respectively.

Mn-CBP possesses high relaxivity required for in vivo imaging of type I collagen deposition. The r_1_ value of Mn-CBP20 at in pH 7.4 solution (10 mM citrate) at 1.4T and 27 °C is or 48.9 mM^−1^s^−1^ per peptide construct (16.3 mM^−1^s^−1^ per Mn^2+^ ion), which is comparable to previously reported r_1_ of EP-3533 recorded under similar, although not identical, conditions, Table 1.

To assess kinetic inertness to Mn^2+^ release, we evaluated Mn^2+^ dissociation kinetics upon challenge of 0.167 mM Mn-CBP20 (0.5 mM Mn^2+^) with 10 mM Zn(OTf)_2_ in the presence of pH 7.4 50 mM Tris/ 18 mM citrate buffer, Figure 2B. The presence of a weak chelator such as citrate is required to maintain homogenous reaction conditions during the course of the measurement, as treatment of Mn-CBP20 with even 1 mol equiv. Zn^2+^ in the absence of chelating buffer precipitates solids from the reaction mixture. Mn^2+^ dissociation was monitored by observing change in r_2_. Under this set of assay conditions, the r_2_ value associated with dissociated Mn^2+^ (16.3 mM^−1^s^−1^) is approximately half that of Mn^2+^ bound within Mn-CBP20. As positive control, we monitored Mn^2+^ dissociation from the kinetically inert complex Mn-CDTA (r_2_ = 5.9 mM^−1^s^−1^). Under these assay conditions, Mn^2+^ dissociates from Mn-CBP20 with half-life (*t*_1/2_) of 22.5 h. Dissociation from Mn-CDTA is slightly faster, with $\left(t_{1/2}\right)$ of 15.5 h.

### Mn-CBP20 enhanced MRI detects collagen deposition in the mouse model of CCl_4_-induced liver fibrosis.

**Figure 3.**
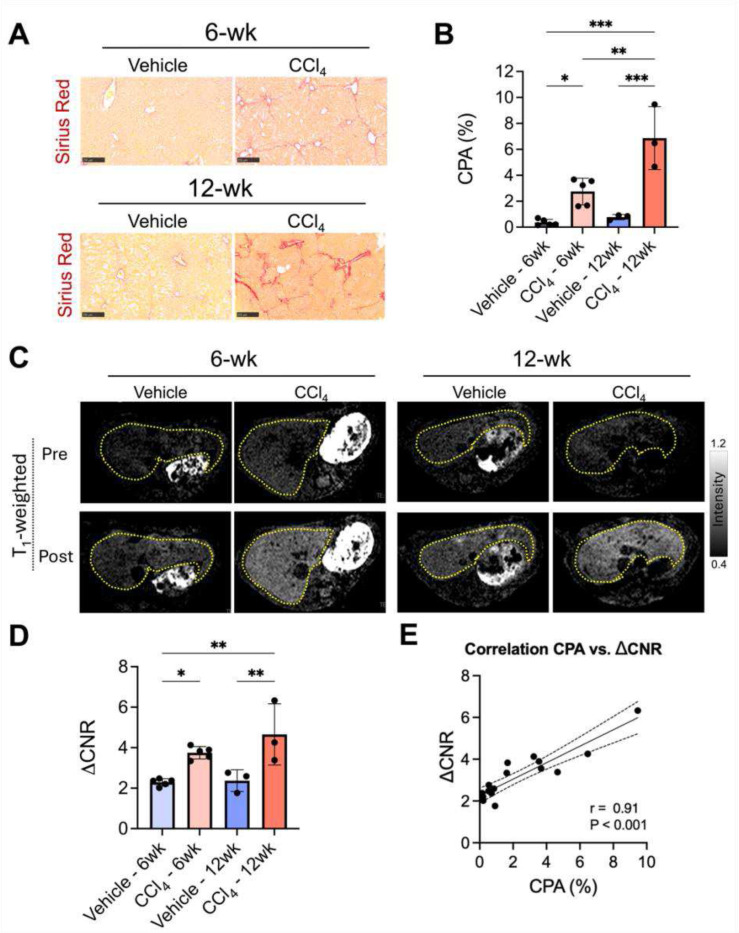
Molecular MRI using Mn-CBP20 in mice treated with carbon tetrachloride (CCl_4_) or vehicle (olive oil) for 6 or 12 weeks to induce liver fibrosis or to serve as controls. **(A)** Representative histologic images of the liver tissues from CCl_4_ and control mice, stained with Sirius Red. **(B)** Total collagen assessed by collagen proportional area (CPA) in Sirius Red stained slides as a fibrosis measure in CCl_4_ and control groups. **(C)** *T*_1_-weighted MRI images acquired before and 45 min post-injection of Mn-CBP20 (10 μmol/kg). **(D)** Comparisons of liver MRI signals in CCl_4_ and control groups, quantified by changes in liver-to-muscle contrast-to-noise ratio (ΔCNR) at 45 min post-injection of Mn-CBP20. One-way ANOVA, *P < 0.05, **P < 0.01, ***P < 0.001, data are shown as means ± SD. **(E)** Correlation plot of ΔCNR (Mn-CBP8) versus %CPA (Sirius Red) in mouse livers showing linear correlation (*r* = 0.91, *P* <0.001).

Sirius Red staining and quantitation of liver collagen show that the percentage collagen proportional area (CPA) was significantly elevated in mice receiving 6-wks CCl_4_ treatment vs vehicle-treated control (2.8 ± 1.0% vs 0.4 ± 0.2%, respectively, P = 0.03), Figure 3A and 3B. CPA further increased after 12 weeks in CCl_4_-treated mice compared to control (6.9 ± 2.4% vs. 0.8 ± 0.2%, respectively, P < 0.001; P = 0.002 vs 6-week CCl_4_ treatment).

Increased liver collagen in CCl_4_-treated mice was reflected in the molecular MR imaging data. Axial T_1_-weighted liver images acquired prior to and 45 min after injection of Mn-CBP20 into mice receiving either 6-week or 12-week regimens of vehicle or CCl_4_ treatment are shown in Figure 3C. The images reflect near complete Mn-CBP20 washout from the liver of vehicle-treated mice but marked delayed liver enhancement in CCl_4_-treated mice. The (pre-post)injection increase in liver-to-muscle contrast-to-noise ratio (ΔCNR) values recorded 45 min after Mn-CBP20 injection were significantly greater in mice receiving 6 weeks of treatment with CCl4 compared to vehicle-treated control (3.8 ± 0.3 vs. 2.3 ± 0.2, respectively, P = 0.03), Figure 3D. Similarly, the ΔCNR values recorded in mice receiving 12 weeks of CCl_4_ treatment were significantly higher than mice receiving 12 weeks of vehicle (4.7 ± 1.5 vs. 2.4 ± 0.5, respectively, P = 0.009), Figure 3D. Furthermore, the ΔCNR values recorded following the injection of Mn-CBP20 correlated tightly with CPA (r = 0.91, P < 0.001), Figure 3E.

**Figure 4.**
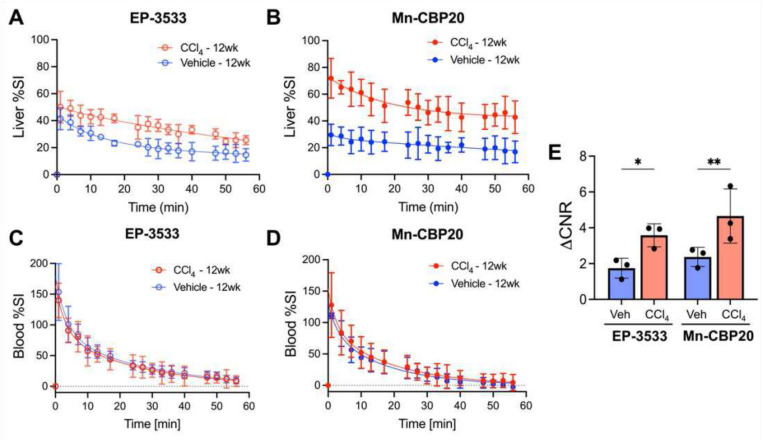
Liver and blood MRI signal dynamics post-injection of collagen-targeted probes EP-3533 or Mn-CBP8. Comparisons of liver percentage signal change (%SI) over time in 12-week CCl_4_ mice and vehicle-treated control mice administered with **(A)** EP-3533 (10 μmol/kg) or **(B)** Mn-CBP8 (10 μmol/kg) (n = 3 / group). Blood %SI over time from the same groups received **(C)** EP-3533 (10 μmol/kg) or **(D)** Mn-CBP8 (10 μmol/kg). (**E**) Both collagen-targeted probes, EP-3533 and Mn-CBP8 induced significantly greater ΔCNR at 45 min post-injection in the livers of 12-wk CCl_4_ mice as compared to controls. One-way ANOVA, *P < 0.05, **P < 0.01, data are shown as means ± SD.

As expected from the in vitro characterization data, Mn-CBP performs similarly to EP-3533 for MR imaging of liver fibrosis. Figures 4A and 4B compare the time course of percentage liver signal change in mice receiving either 12 weeks of treatment with CCl_4_ or vehicle control following injection of Mn-CBP20 or EP-3533, respectively. Both probes differentially generated greater liver enhancement in CCl_4_ treated mice that is sustained from 1 min post-injection up to at least 55 min post-injection. There was no difference in blood half-life in the CCl_4_ and vehicle-treated mice for EP-3533 (17.8 ± 2.5 min vs. 18.9 ± 2.6 min, P = 0.86) or Mn-CBP20 (13.5 ± 1.5 min vs. 12.2 ± 2.4 min, P = 0.76), as shown in Figures 4C and 4D. Separate comparisons of ΔCNR generated 45 min after injection of either probe in CCl_4_ vs. vehicle-treated mice indicate that MnCBP-20 offers comparable sensitivity to EP-3533 for detection of liver fibrosis, Figure 4E.

### Mn-CBP20 enhanced MRI detects liver fibrosis in a nutritional mouse model of MASH.

**Figure 5.**
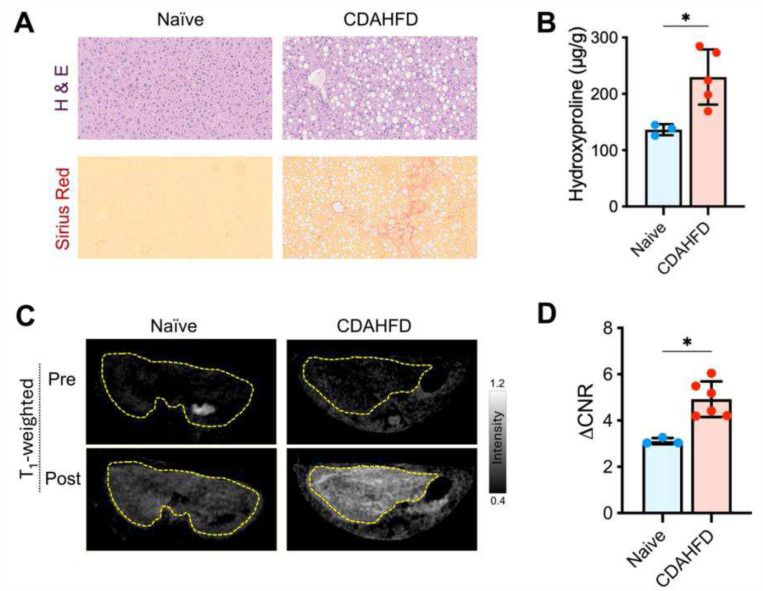
Molecular MRI using Mn-CBP20 in mice fed with choline-deficient L-amino acid-defined high-fat diet (CDAHFD) or standard chow (naïve) for 6 weeks. **(A)** Representative histologic images of the liver tissues from CDAHFD and control mice, stained with H&E and Sirius Red, respectively. **(B)** Total collagen assessed by hydroxyproline assay as a fibrosis measure in CDAHFD and naïve control groups. Unpaired Student’s t-test, *P < 0.05, data are shown as means ± SD. **(C)** *T*_1_-weighted MRI images acquired before and 40 min post-injection of Mn-CBP20 (10 μmol/kg). **(D)** Comparisons of liver ΔCNR at 35–45 min post-injection of Mn-CBP20.

We further evaluated Mn-CBP20 molecular MRI as a marker of liver fibrosis using age-matched mice receiving either a 6-week regimen of CDAHFD or standard chow. CDAHFD results in steatohepatitis that recapitulates salient features of human MASH including liver fibrosis, as evidenced by the histologic data shown in Figure 5A where severe steatosis and increased liver collagen evident in specimens stained with H&E and Sirius Red, respectively. HPLC quantitation of liver hydroxyproline content, an amino acid that is highly abundant in type 1 collagen, indicates that levels are significantly higher in mice fed CDAHFD vs standard diet (638 ± 135 μg/g vs. 196 ± 61 μg/g, respectively, P = 0.002) also consistent with liver fibrosis, Figure 5B.

Figure 5C shows axial T_1_-weighted images recorded in mice fed CDAHFD and standard diet prior to and 45 min after injection of Mn-CBP. Markedly stronger liver enhancement is observed in CDAHFD, consistent with increased liver collagen content. The effect is also reflected in quantitation of liver enhancement, with significantly greater ΔCNR in mice fed CDAHFD vs. standard diet (4.9 ± 0.8 vs. 3.1 ± 0., P = 0.02), Figure 5D.

### Mn biodistribution.

^52^Mn biodistribution was evaluated following injection of [^52^Mn]Mn-CBP20 mixed with ^nat^Mn-CBP20 at the same mass dose as used for MRI. This radio-labelling approach enables us to tomographically visualize whole-body biodistribution of administered Mn in vivo using PET, and to definitively discern administered Mn from endogenous Mn in tissues.

**Figure 6.**
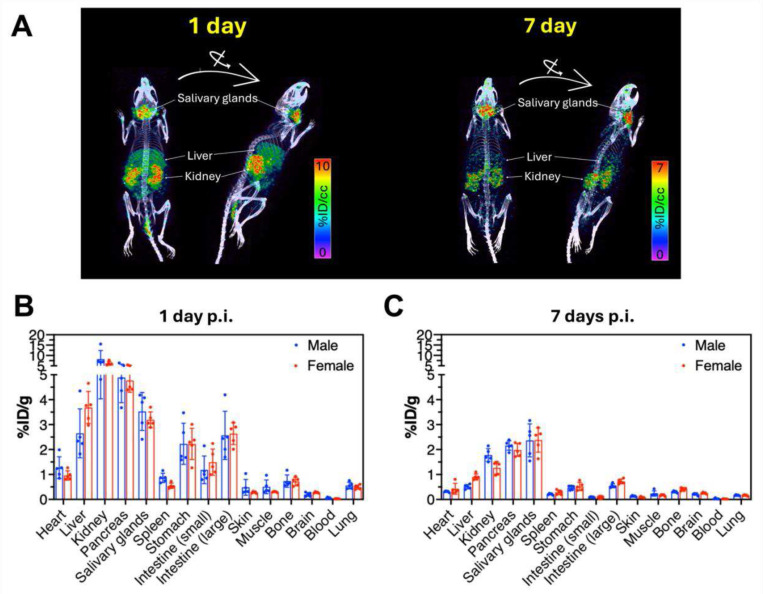
Biodistribution of Mn-CBP20 probe in healthy control mice. **(A)** Whole body PET-CT(color scale) images of normal mice imaged at 1 day and 7 days post-injection of [^52^Mn]Mn-CBP20. PET imaging is reported as percentage injected dose per cubic centimeter (% ID/cc). *Ex vivo* biodistribution of [^52^Mn]Mn-CBP20 at **(B)** 1 day and **(C)** 7 days p.i., expressed as percent injected dose per gram of tissue (%ID/g) in various organs and tissue. n = 10 mice (5 males and 5 females) / timepoint. Data are shown as means ± SD.

Whole body PET-CT images recorded 1 and 7 days after [^52^Mn]Mn-CBP20 injection are shown in Figure 6A and ex vivo ^52^Mn biodistribution data are shown in Figure 6B and 6C and also tabulated in Supplementary Tables 1 and 2. The highest concentration of ^52^Mn 1 day after injection is found in the kidneys, consistent with the expected renal elimination of the compound. Substantial activity is also found in the liver and organs of the gastrointestinal tract which could be related to partial elimination of the probe via the hepatobiliary path but might also be related to elimination of any dechelated ^52^Mn. Other tissues with relatively high concentrations of ^52^Mn 1 day after [^52^Mn]Mn-CBP20 injection include the pancreas, heart, and salivary glands. By day 7, residual activity in the kidney, liver, gastrointestinal tract, pancreas, and heart decreased substantially, but relatively high levels of residual activity in the salivary glands persisted. In prior studies to evaluate ^52^Mn biodistribution after injection of the low molecular Mn complex Mn-DPDP in rats, high and persistent ^52^Mn uptake in the salivary glands relative to other tissues was also observed.^26^

## Discussion

Liver fibrosis is a hallmark feature of progressive CLD, and the fibrosis stage is a predictive marker for poor outcomes such as liver cancer or end-stage liver disease.^30–32^ Patients with advanced stage liver fibrosis face a high risk of death from CLD and few available treatment options. Patients with mild to moderate fibrosis stand a better chance of benefitting from interventions to halt or even reverse CLD progression.^33^ However, moderate fibrosis often occurs in the absence of clinical signs and too often remains undetected until progression to a more advanced stage. Patients suffering from progressive CLD could benefit immensely from noninvasive tools to diagnose, stage, and track the progression or resolution of moderate liver fibrosis.

A prior study using a rat model of bile duct ligation indicated that molecular MR imaging using the experimental type I targeted probe EP-3533 in rats provided sensitivity to detect mild and moderate liver fibrosis that could not be detected using MR elastography.^14^ Although the technology is promising, challenges to developing EP-3533 for clinical imaging are anticipated. EP-3533 uses Gd as the MR signal-generating component. Concerns over the presence of residual Gd that is retained by patients who receive contrast-enhanced MRI scans have been widely publicized and contributed to a challenging regulatory landscape for the development of new Gd-based imaging probes. Mn-based imaging probes offer a potentially viable alternative to Gd-based imaging probes. The Mn^2+^ ion is a very effective T_1_-relaxation agent and Mn^2+^ is an endogenously present nutritional element.^34^ In recent years, Mn-based imaging probes have advanced to evaluation in clinical trials.^35,36^

In this study, we synthesized and evaluated a Mn-based probe targeted to type I collagen termed Mn-CBP20. In vitro characterization showed that Mn-CBP20 binds to human type I collagen with comparable affinity to EP-3533, and also exhibits comparable relaxivity. Direct comparison of Mn-CBP20 to EP-3533 in a mouse model of CCl4-induced liver fibrosis indicates that Mn-CBP20 is equally effective to EP-3533 for the detection of liver collagen deposition, and thus suggests that Mn-CBP20 may offer similar advantage over current clinical imaging for detection of mild to moderate liver fibrosis. Mn-CBP20 was also effective for the detection of liver fibrosis in a CDAHFD mouse model of MASH, underscoring the potential use of what is both the most prevalent and most rapidly increasing CLD.

There are limitations to the study. While the straightforward synthesis of Mn-CBP20 highlights the potential for low cost of goods manufacture, synthetic activities for this study focused narrowly on obtaining small amounts of compound for preliminary evaluation, rather than on process optimization. MR imaging data obtained using Mn-CBP20 was evaluated in the context of liver collagen content determined through ex vivo biochemical assays, which underscores collagen specificity and the potential to quantify liver collagen deposition. However, MR imaging using Mn-CBP20 was not evaluated in the context of the histologically assigned fibrosis stage, and our study did not compare against currently available serum biomarker panels or clinical imaging that can generally reliably detect advanced fibrosis. We performed imaging studies only in male mice which have been reported to have a stronger fibrotic response than females^37^. Future studies should include female animals. We used EP-3533 as a positive control in the imaging studies and animals with no fibrosis as negative controls. A negative control probe was not used here but we have previously shown that untargeted probes do not detect liver fibrosis compared to EP-3533^38^.

In conclusion, Mn-CBP20 represents a promising candidate that merits further evaluation and development for molecular imaging of liver fibrosis.

## Figures and Tables

**Table 1.** Comparisons of Mn-CBP20 and EP-3533 physical properties relevant to collagen molecular imaging.

	$K_{\text{d}}\;\left(\text{μM}\right)$ ^a^	per molecule r_1_ (mM^−1^s^−1^)	per metal ion r_1_ (mM^−1^s^−1^)	per molecule r_2_ (mM^−1^s^−1^)	per metal ion r_2_ (mM^−1^s^−1^)
**Mn-CBP20**	9.5	48.9^b^	16.3^b^	117.9^b^	39.3^b^
**EP-3533**	5.5	48.3^c^	16.1^c^	77.7^c^	25.9^c^

a Kd determined in the presence of human type I collagen

b 1.41T and 27 °C, pH 7.4 50 mM Tris: 10 mM citrate.

c 1.41T and 37 °C, PBS.
